# The Science of Temperance

**Published:** 1878-02

**Authors:** 


### THE SCIENCE OF TEMPERANCE.

In a late lecture with this title, Dr. B. W. Richardson, of London, made the following points:—First: That the substance now called alcohol, and which had been so called for some three centuries, could not be considered as a food, as most people supposed—standing alone in the world as some thing which was to be taken as if it were a food. Second: That common alcohol was, therefore, not a special gift sent to them to be used as a food, any more than the other chemical bodies coming under the head "Alcohol." Third: That when, as physiologists and biologists, they looked on the construction of the animal kingdom, and considered how it was made up of certain fluids and solids, they were struck with the fact that there was no provision whatever made for the use of such an agent as alcohol. Nature had produced the organization simply one of fluid, and that fluid was water. Fourth: That ethylic alcohol acted on the bodies of men and animals in the same manner as other chemical substances. It did not act after the manner of a food at all, but produced effects which were phenomenal in their character. He found that a fatal dose meant a proportion of a drachm of fluid, to the pound weight of the warm-blooded animals. In a man weighing 120 lbs., a dose of fifteen ounces would certainly be fatal unless scientific means averted death. The lecturer then graphically described the phenomenal effects of various doses of alcohol on the organism, and remarked in conclusion, that if alcohol did anything that was of use in the animal organization, it was only in the first stage of this action.

### THE STUDENT'S HYSTERIA.

In a paper on hysteria, which received a prize at the Physical Society of Guy's Hospital this year, Mr. P. Horrocks writes:—

"During the fortnight following the death of the late Napoleon, Sir James Paget was consulted for stone in the bladder, by no less than four gentlemen who had nothing the matter with them. And this leads me to speak of a form of hysteria which is frequent in males, and perhaps more so in our own profession than in any other class of people. How many students are there of one year's standing or more, in this hospital or any other, who have not imagined, and really become convinced, that they were suffering from some disease generally a fatal disease? I, myself, must confess that I have, since coming to Guy's, been thoroughly convinced that my heart was diseased. After a time, however, I felt that I was laboring under a great delusion; it was not my heart, after all, it must be my lungs. I remember listening with breathless attention to Dr. Habershon, as he lectured on phthisis, for I was so convinced that my chest was effected. that I had not, at that time, called up sufficient courage to read it in books, for fear of finding out, without any doubt, that I was a doomed man. One thing, however, I could not get over, and that was that phthisical patients lose their appetites. I have never had that symptom yet, and so, after all, I may only have been suffering from mental delusion. I am not alonein this kind of thing; scores of students consult, yearly medical men, for complaints of which they have not a single symptom. Ask any of our staff; they have had ample experience, and will fully bear out what I have stated."

